# The Implementation and Evaluation of a Media Literacy Intervention About PAES Use in Sport Science Students

**DOI:** 10.3389/fpsyg.2020.00368

**Published:** 2020-03-24

**Authors:** Luca Mallia, Andrea Chirico, Arnaldo Zelli, Federica Galli, Tommaso Palombi, Laura Bortoli, Cristiana Conti, Pierluigi Diotaiuti, Claudio Robazza, Federico Schena, Francesca Vitali, Thomas Zandonai, Fabio Lucidi

**Affiliations:** ^1^Department of Movement, Human and Health Sciences, Foro Italico University of Rome, Rome, Italy; ^2^Department of Social and Developmental Psychology, Sapienza University of Rome, Rome, Italy; ^3^BIND-Behavioral Imaging and Neural Dynamics Center, Department of Medicine and Aging Sciences, G. d’Annunzio University of Chieti-Pescara, Chieti, Italy; ^4^Laboratory of Epidemiology, Physical Activity and Lifestyles, Department of Human Sciences, Society and Health, University of Cassino and Southern Lazio, Cassino, Italy; ^5^Department of Neurosciences, Biomedicine and Movement Sciences, University of Verona, Verona, Italy; ^6^Mind, Brain and Behavior Research Center, Department of Experimental Psychology, University of Granada, Granada, Spain

**Keywords:** performance and appearance enhancement substance use, media literacy intervention, attitudes, sport sciences students, self-efficacy

## Abstract

With respect to both competitive and amateur/fitness sports, media may strongly influence young people’s opinions and behaviors concerning the use of PAES (*Performance and Appearance Enhancing Substances*). The present investigation addressed this topic by focusing on sport sciences students’ beliefs concerning the possible role of media related to the implementation and evaluation of a PAES-focus media literacy intervention conducted with sport science students. This study relied on a sample of 521 students (attrition rate 10.3%) (45.1% female, mean age = 22.6, SD = 2.20), which provided baseline data on students’ levels of media literacy concerning the use of PAES (i.e. “descriptive sample”), and a sample of 248 students, who participated in and provided data on the media literacy intervention. This latter sample included a group of 128 students (44.5% female, mean age = 23.03, SD = 3.76) who actively participated in the intervention (i.e. “intervention group”), and a group of 120 students who did not (i.e. “control group”, 53.3% female, mean age = 22.25, SD = 2.47). All students filled out media literacy questionnaires targeting students’ awareness of media influence, their views about the realism of media content, their sense of confidence in dealing with media messages, and their positive attitudes toward PAES use. Analyses of questionnaire data showed that students are relatively aware of media influence on people’s views and behaviors with respect to PAES use. At the same time, students also believed that young people do not consider media as “realistic sources” of information; nonetheless, they also did not consider themselves entirely capable of dealing effectively with media messages. With respect to the intervention, students overall appreciated and greatly welcomed the educational program on media literacy, and the analyses of intervention data across intervention and control groups showed that key media literacy variables changed over time, attesting to the overall effectiveness of the intervention.

## Introduction

### The Use of Substances Guided by Performance and Appearance Enhancement

The use of Performance and Appearance Enhancing Substances (PAES) that are clearly subject to legal sanctions and often labeled “controlled”^1^ has steadily increased among young athletes and exercisers in recent times (Mallia et al., 2013). A more recent European study of a large sample of young amateur athletes and exercisers has shown that nearly one out of five participants has had experience with controlled PAES in the past (Lazuras et al., 2017).

Generally, prevalence data on controlled PAES reveals that males are more at risk than females, and that older adolescents used PAES more often than younger adolescents (e.g. Yesalis and Bahrke, 2000; Johnston et al., 2007). Additional scientific evidence also attests that the use of controlled PAES, such as anabolic–androgenic steroids (AAS), is present among high-level athletes of many types of sport (Dunn and White, 2011; Mallia et al., 2013) and among non-athletes (e.g. Pope et al., 2014; Sandvik et al., 2018).

Prevalence rates vary across studies. For instance, Sandvik et al. (2018) have shown that about 1.3% of nearly 80,000 Norwegian adolescents have repoted using AAS. Kargarfard et al. (2015) have indicated that 8% of university students have used AASs in the past and that 6% of the sample reported using AASs at the time of the study.

The use of controlled PAES has important health implications, since it tends to be associated with a wide range of adverse mental and physical health problems, both in adolescent athletes and non-athletes (e.g. Bird et al., 2016). Furthermore, the use of controlled PAES among athletes practicing elite sports is perceived as a clear violation of sports rules. Indeed, their use is strictly regulated by sports authorities governing competitive sports (e.g. WADA and NADOs). In contrast, the legal and social sanctions associated with the use of controlled PAES among those practicing fitness and recreational sports are less clear, a fact that tends to induce greater acceptance in society (Lazuras et al., 2017).

An increase in prevalence rates has also been seen in PAES often labeled as “uncontrolled”^2^. This increase appears among sportsmen and athletes at different competitive levels (elite, amateur, and recreational) and of different ages (Knapik et al., 2016). As in the case of “controlled” PAES, this increase has raised some concerns with respect to their long-term health consequences (e.g. Metzl et al., 2001; Biggs et al., 2017).

The reasons that seem to guide the use of “uncontrolled” PAES generally fall under two distinct rubrics. There is the possibility that users see uncontrolled PAES as a “safe alternative” to the use of prohibited/illegal substances (e.g. Petróczi et al., 2011). Alternatively, there exists the “*gateway*” hypothesis, that is, the possibility that the use of uncontrolled substances (e.g. supplements) represents a “gateway” to legally and socially sanctioned substance use (e.g. steroids – see for instance, Lucidi et al., 2008; Zelli et al., 2010; Mallia et al., 2013; Ntoumanis et al., 2014).

### The “Power of Media” on Health-Related Risk Behaviors

Media, such as newspapers, radio stations, TV, and the internet (e.g. websites, social media, YouTube, and message boards) today represent the primary channels for information flow. With this in mind, media may give PAES users their points of view on the world and on events. According to the *agenda-setting theory* (McCombs and Shaw, 1972), the emphasis of media on specific and targeted issues, events, and contents, tends to guide and direct public attention (i.e. *thematic agenda*) and to shape people’s views (i.e. *attribute agenda*). Indeed, media may influence people’s social-cognitive information processing, by emphasizing and endorsing particular views of reality, with the net result of guiding and suggesting particular modes of action or behavior (e.g. Gamson et al., 1992; Gamson, 2004).

This scientific focus on the role of media has found fertile ground in the context of risky health-related behaviors, such as substance use/abuse (e.g. Strasburger et al., 2010). For instance, media messages about alcohol and tobacco use tend to influence or guide young people’s beliefs, attitudes, and behaviors concerning these substances (e.g. Austin and Knaus, 2000; Zucker et al., 2008; Scull et al., 2010, 2014).

This focus is also of urgent interest in the sport domain, with particular efforts targerting PAES use (e.g. Agulló Calatayud et al., 2014; Borel-Hänni et al., 2019; Richardson et al., 2019). Scientific work has also looked at “social networking” and the ways peers’ posted contents, unregulated advertising, and information shared across social media may influence adolescents’ positive attitudes about substance use (e.g. Scull et al., 2010, 2014). For instance, media may portray PAES use as a positive and valid way to reach certain personal goals (e.g. the desire to enhance performance and/or physical appearance) and thus promote positive attitudes among adolescents (Carstairs, 2003). On a different account, some scholars (Brown et al., 2003) have instead pointed out that the global media exposure to popular sports events has forged sport “icons” whose (possibly negative) health-related behaviors may greatly influence their fans and supporters’ behaviors.

### Media Literacy Interventions Contrasting “Media Power”

With these considerations in mind, it is important to identify educational strategies and programs promoting a correct analysis and management of media messages and, indirectly, the endorsement of positive attitudes and health-promoting behaviors (Kupersmidt et al., 2010). These programs fall under the rubric of “media literacy training”. They typically focus on young people’s ability to use a media product (e.g. advertisements, blogs, websites), and to identify its intended target consumers, its messages, the producer’s message, and who will benefit from a full (and behavioral) endorsement of the media communication that has been used to promote the product.

According to Potter’s (2004), cognitive approach media literacy skills rely on three distinct and related cognitive processes: a process of *knowledge acquisition* concerning, for instance, media contents and media effects; a process of *correct cognitive elaboration*, typically focusing on the steps of analysis, evaluation, and abstraction; and a process of *cognitive re-frame* focusing on personal awareness and goals and one’s capacity to minimize the possible detrimental effects of media messages.

This theoretical framework suggests that, at least in young people (see Kupersmidt et al., 2010), media literacy should contribute to the acquisition of one’s skills in evaluating critically and correctly media messages and in resisting the behavioral pressure these messages may ensue. Evidence from existing literature is consistent with this general notion, and several media literacy education programs focusing on health offered young people the opportunity to critically examine media messages targeting different unhealthy behaviors, such as the abuse of alcohol (e.g. Austin and Johnson, 1997), tobacco (e.g. Banerjee and Greene, 2006) or drugs (e.g. Eisen, 2002), sexual behaviors (e.g. Pinkleton et al., 2008), or weight management (e.g. Wilksch and Wade, 2009).

There exists considerable research on the efficacy of media literacy education programs targeting health behaviors. For instance, the meta-analysis by Se-Hoon et al. (2012) scrutinized 51 media literacy interventions, and its findings overall supported these programs’efficacy with respect to outcomes such as media knowledge, criticism of media messages, their realism and influence, views about media messages, attitudes, self-efficacy, and health behaviors (Se-Hoon et al., 2012).

Likewise, Vahedi et al. (2018) more recent meta-analysis focused on media literacy interventions that distinctly targeted media literacy skills and young people’s attitudes and intentions toward unhealthy behaviors. Overall, its findings supported positive and statistically significant effects on both accounts and highlighted that interventions tend to moderate the effects on attitudes and intentions and have no such moderation with respect to media literacy skills (Vahedi et al., 2018). However, these meta-analysis findings need to be interpreted with some caution, as they might be affected by methodological and/or analytical biases. More generally, as Potter and Thai (2019) pointed out in their very recent review, the literature on media literacy educational programs tends to show problems of definition and operationalization of relevant constructs (i.e. content validity), as well as poor correspondence between initial measurement designs and actual data (i.e. face validity).

### A Doping-Related Media Literacy Intervention

Within the framework of the scientific literature on media literacy, Lucidi et al. (2017) offered the first effort in the design, implementation, and evaluation of a media literacy intervention focusing specifically on PAES use in sport settings. The goals of this educational program were in line with efforts in other domains of substance use, and the intervention focused on youth and on the possibility of assisting them in their views and evaluation of their performance and/or esthetic goals.

The findings of Lucidi et al. (2017) investigation were encouraging and overall suggested that high school students benefitted from the media literacy intervention, as evidenced by a decrease in students’ positive attitudes toward doping use and in their reported use of uncontrolled PAES (e.g. supplements).

### The Present Investigation

The present study had two main objectives. The first was to establish, among a large sample of university sport sciences students, the psychometric characteristics of a set of media literacy measures concerning beliefs about media and their possible role in soliciting PAES use. The second objective was to implement and to evaluate the effects of a doping-related media literacy intervention in a second sample of university sport sciences students. The media literacy intervention followed and extended the educational program implemented by Lucidi et al. (2017). The study overall focused on university sport sciences students, as they might be at risk for doping use (see Thevis et al., 2008) and, at the same time, might in their professional life have a critical educational role (e.g. as coaches or trainers) toward young athletes and non-athletes.

## Materials and Methods

### Participants and Procedures

Students, distributed evenly across four Italian university degree programs in sport sciences (i.e. Rome, Verona, Chieti, and Cassino), were initially contacted and fully informed about the general aims of the study. The study was approved by the Ethics Review Board of the Department of Social and Developmental Psychology, “Sapienza” University of Rome. The study relied on written consent for participation, and recruitment procedures led to the selection of two distinct samples.

The first sample initially comprised 581 university sport sciences students, and 521 of them gave their consent for participation (nearly 90%). This sample (45.1% females, mean age = 22.6, SD = 2.20) practiced fitness activities (33.0%), individual (33.2%) and team sports (33.8%), and provided data on the set of media literacy measures mentioned earlier (i.e. “descriptive sample”).

The second sample comprised of 248 sport sciences university students (i.e. “evaluation sample”), and all of them gave their consent to participate to a media literacy intervention study. In particular, a group of 128 students (44.5% female, mean _age_ = 23.03, SD = 3.76) actively participated in the intervention (i.e. “intervention group”), whereas a group of 120 students (53.3% female, mean _age_ = 22.25, SD = 2.47) did not (i.e. “control group”).

### Assessment

Students in both samples provided data on the set of media literacy measures mentioned earlier. In particular, while students in the “descriptive sample” provided one-time data on the measures, students in the “evaluation sample” provided data on the measures twice, that is, either before and after the intervention sessions (i.e. students who actively participated to the intervention) or within a similar timeframe (i.e. students in the “control” group).

Media literacy measures focused on the following key variables:

1. Students’ awareness of the possible influence media might have on beliefs and behaviors concerning PAES use in sport. The measurement of this variable was an adaptation of the “Awareness of Media Influence Scale” (Pinkleton et al., 2008), and it comprised three separate sets of four items evaluating, respectively, the influence of social media, TV, and newspapers. A sample item was “Social Media/TV/Newspaper messages affect the way young people think about the use of substances to enhance their physical appearance”. Students rated items using a 7-point response scale ranging from 1 (“Totally disagree”) to 7 (“Totally agree”).
2. Students’ perception of media realism. The measurement of this variable was an adaptation of the Austin et al. (2005) scale with respect to four different types of media (i.e. TV, Newspaper, Web sites, Social Media) and, for each type of media, it comprised a set of four items. A sample item was “TV/Newspaper/Websites/Social Media are realistic sources of information for how people my age act”. Students rated items on a response scale ranging from 1 (“Never”) to 7 (“Always”).
3. Students’ sense of personal confidence in dealing with media messages (i.e. perceived self-efficacy). The measurement of this variable relied on the 3-item scale developed by Austin et al. (2005), and a sample item of this scale was “I have ideas about how I can use media to affect whether other teenagers use substances to enhance their performance or physical appearance”. Students rated items on a 7-point response scale ranging from 1 (“Totally disagree”) to 7 (“Totally agree”).
4. Students’ positive attitudes toward PAES use. The measurement of this variable relied on a validated set of items (Lucidi et al., 2008, 2013, 2017; Zelli et al., 2010; Mallia et al., 2013) which asked students to express to what extent their “use of PAES would be. useless/useful, foolish/wise, undesirable/desirable, negative/positive, harmful/beneficial, and advantageous/disadvantageous”. Students rated each of these dimensions using a 5-points scale.

### Intervention and Its Implementation

The intervention design overall followed the same structure of Lucidi et al. (2017) intervention with high school students. The intervention comprised of twelve 90-min sessions and primarily relied on four experts (a sport psychologist, a communication expert, a pharmacologist, and a retired top-level athlete), each of whom led two intervention sessions with the sport science students across the four university sites of the study.

These experts focused on media messages concerning PAES use in sport from their own expertise viewpoint. In the first two sessions, the sport psychologist focused on the ways mental strategies may help sport science students in handling and evaluating media messages, as well as on students’ awareness of their own personal goals and capacity to counteract temptations toward PAES use. Similarly, the communication expert led the third and fourth sessions by addressing the role media messages may have in promoting dysfunctional beliefs about sport and PAES use via, for instance, an emphasis on unrealistic objectives, such as an ideal body shape or a “heroic” performance.

The pharmacologist led the fifth and sixth sessions by providing scientific and correct information on PAES and their possible effects, thus counteracting the so-called “dark side” of media messages, that often exclusively highlight the (always only presumed) positive effects of PAES. Finally, the (retired) high-level athlete led the last two sessions by primarily focusing on the moral, ethical, and health implications of PAES use and on the ways media may offer arguments to minimize or to ignore these sorts of issues. During the first eight sessions, participants were actively encouraged to share their personal views, discuss issues among peers, and/or work together in small groups for practical activities that were designed to reinforce key intervention topics or contents.

Finally, following recommendations from other authors (see, for instance, Banerjee and Greene, 2006), sessions nine through twelve focused on guding sport science students, divided into small groups, through fully autonomous activities designed to reinforce the intervention’s goals and topics. Four psychologists with expertise in communication (one for each university site) acted as tutors and supersvisors of these last four sessions. The specific protocol for these sessions was outlined in a training manual in order to standardize the practical activities of the small groups as much as possibile.

In particular, the supervisors invited students to find existing messages concerning PAES use in the media and to analyze them from different points of view (e.g. content, purpose, target, etc.). The key task of these four sessions was the design of an “awareness-raising campaign” or a media message that would explicitly take a position against PAES use. Overall, the program was greatly welcomed by participants, since the rate of the students’ attendance across the sessions was very high (93%).

### Data Analysis

The first set of analyses focused on the media literacy measures and their reliability (i.e. Cronbach’s Alpha) and distribution characteristics (i.e. means, skewness, and kurtosis). These analyses were conducted on the measurement data provided by students in the “descriptive” sample.

The second set of analyses instead focused on the media literacy intervention and the assessement of its efficacy. In particular, two separate repeated measures Group by Time MANOVAs were first performed to assess whether university students who participated in the intervention sessions reported, as compared to their control group counterparts, significant changes across pretest and posttest measurements, respectively, in their awareness of media influence and the level of realism media messages have. Since these two variables were assessed separately across different types of media (e.g. TV, web, newspapers), the analyses followed a MANOVA design. In contrast, two repeated measures Group by Time ANOVAs separately evaluated possible changes over time in students’ self-efficacy and positive attitudes toward PAES.

## Results

### Descriptive and Psychometric Characteristics of Media Literacy Measures

As to the first set of analyses, Table 1 shows the reliability (alpha) coefficients of all the key measurement scales. Coefficients were relatively high and ranged from 0.71 to 0.96, thus overall supporting the internal consistency of the item sets. Furthermore, the distribution indices of skewness and kurtosis indicated that measurements had overall normal-like data distributions.

**TABLE 1:** Descriptive statistics of the key measures of the study derived from the descriptive sample (*n* = 521).

Key media literacy variables	No. items	Response range	Mean (SD)	Skewness	Kurtosis	Cronbach’s Alpha
**Awareness of …**						
Social media influence	4	1–7	4.98 (1.31)	0.08	−0.54	0.92
Television influence	4	1–7	4.51 (1.39)	−0.47	−0.16	0.94
Newspapers Influence	4	1–7	4.22 (1.46)	−0.52	−0.11	0.96
**Perceived realism of…**						
Television	4	1–7	3.32 (1.06)	0.05	−0.15	0.71
Newspapers	4	1–7	3.42 (1.08)	0.06	−0.14	0.76
Web sites	4	1–7	3.79 (1.25)	−0.02	−0.09	0.78
Social Media	4	1–7	3.74 (1.29)	−0.08	−0.21	0.77
Perceived self-efficacy in dealing with media	3	1–7	3.72 (1.35)	0.12	−0.55	0.76
Positive attitudes toward PAES use	6	1–5	2.05 (0.83)	0.16	−1.27	0.81

Table 1 also shows the descriptions of all measurements. Should one consider the theoretical mean level of scales as a comparison term, Table 1 shows that university students reported relatively high mean levels of awareness about media influence on young peoples’ beliefs and behaviors related to PAES use, and this held for different types of media. Sport sciences students also reported mean levels of perceived media realism that were in line with the theoretical mean level of the scale. That is, students on average believed that media (television, newspapers, websites, and social media) represent ”realistic sources” for the opinions and choices young people hold. Similarly, sport sciences university students on average felt somewhat confident that they would deal effectively with media meassages and pressure targeting a positive endorsement of PAES use.

### The Assessment of Media Literacy Intervention

#### Awareness of Media as Sources of Influence

At a multivariate level, the results showed no significant Group by Time interaction effect on students’ awareness of media influence (Wilks’ Lamba _(__3_,_237__)_ = 0.728; *p* = 0.536; partial eta square = 0.009), whereas there was a significant Time main effect (Wilks’ Lamba _(__3_,_237__)_ = 7.766; *p* < 0.001; partial eta square = 0.090).

These multivariate effects were more carefully examined at the univariate level of pairwise comparisons. At this level, there were time effects (i.e. differences between pretest and posttest scores) across groups. In particular, the mean levels of awareness of media influence significantly decreased among control group students with respect to both newspapers [*F*_(__1_,_239__)_ = 11.55, *p* = 0.001; partial eta square = 0.046; Mean _(time__1__)_ = 4.37; SD _(time__1__)_ = 1.60, Mean _(time__2__)_ = 3.87; SD _(time__2__)_ = 1.62] and TV [*F*_(__1_,_239__)_ = 11.85, *p* = 0.001; partial eta square = 0.047; Mean _(time__1__)_ = 4.70; SD _(time__1__)_ = 1.49, Mean _(time__2__)_ = 4.25; SD _(time__2__)_ = 1.44], whereas no such decreases in awareness of media influence emerged among intervention group students on either newspapers [*F*_(__1_,_239__)_ = 2.20, *p* = 0.14; partial eta square = 0.009; Mean _(time__1__)_ = 4.20; SD _(time__1__)_ = 1.32, Mean _(time__2__)_ = 3.99; SD _(time__2__)_ = 1.28] or TV [*F*_(__1_,_239__)_ = 3.38, *p* = 0.067; partial eta square = 0.014; Mean _(time__1__)_ = 4.58; SD _(time__1__)_ = 1.32, Mean _(time__2__)_ = 4.35; SD _(time__2__)_ = 1.16].

Finally, results showed a different pattern when the analyses were concerned with students’ awareness of the influence that social media may have. In this case, pairwise comparisons showed significant Time effects, as awareness scores decreased over time in both control group students [*F*_(__1_,_239__)_ = 7.44, *p* = 0.007; partial eta square = 0.030; Mean _(time__1__)_ = 5.19; SD _(time__1__)_ = 1.42, Mean _(time__2__)_ = 4.72; SD _(time__2__)_ = 1.39] and intervention group students [*F*_(__1_,_239__)_ = 13.17, *p* < 0.001; partial eta square = 0.052; Mean _(time__1__)_ = 5.13; SD _(time__1__)_ = 1.26, Mean _(time__2__)_ = 4.80; SD _(time__2__)_ = 1.14].

#### Perceived Realism of Media as Sources of Influence

The MANOVA results concerning students’ perceived level of media realism showed patterns that were similar to those of awareness of media influence.

There were no multivariate significant Group by Time effects [Wilks’ Lamba _(__4_, _236__)_ = 1.44; *p* = 0.220; partial eta square = 0.024]. Nonetheless, pairewise comparisons qualified this null finding. There was a statistically significant Group by Time effect on students’ perceived realism of newspapers [*F*_(__1_, _239__)_ = 4.96; *p* = 0.027; partial eta square = 0.02], whereas the Group by Time effect on students’ perceived realism of TV approached statistical significance [*F*_(__1_, _239__)_ = 3.05; *p* = 0.082; partial eta square = 0.013].

**FIGURE 1:**
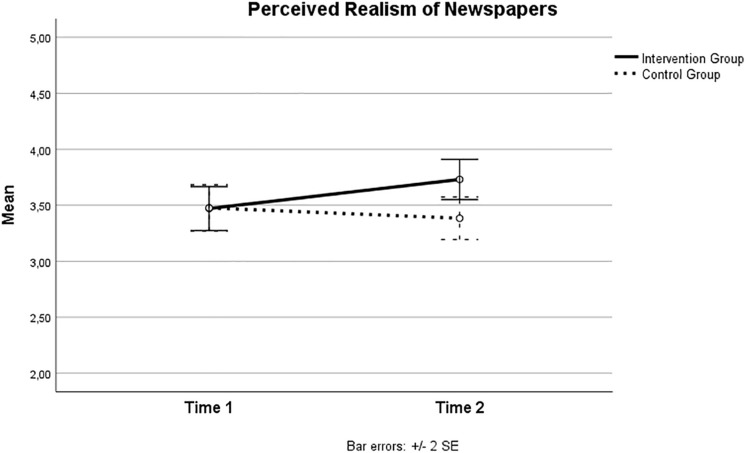
Perceived realism of newspaper across time in intervention and control groups.

The statistically significant Group by Time effect concerning students’ perceived realism of newspapers is depicted in Figure 1. Consistent with the figure, univariate results showed that realism of newspapers as possible sources for young people’s beliefs and choices increased over time among intervention group students [*F*_(__1_,_239__)_ = 5.72; *p* = 0.018; partial eta square = 0.023]. In contrast, newspapers realism scores among control group students did not change over time [*F*_(__1_,_239__)_ = 0.64; *p* = 0.423; partial eta square = 0.003].

In the case of the close-to-siginificance Group by Time interaction concerning TV realism, the results were very similar to those of newspaper realism, albeit non-statistically significant. Intervention group students’ TV realism scores also increased over time [*F*_(__1_,_239__)_ = 4.21; *p* = 0.04; partial eta square = 0.017; Mean _(time__1__)_ = 3.48; SD _(time__1__)_ = 1.01; and Mean _(time__2__)_ = 3.67; SD _(time__2__)_ = 0.90], whereas control group students’ TV realism scores still showed no significant change over time [*F*_(__1_,_239__)_ = 0.215; *p* = 0.643; partial eta square = 0.001; Mean _(time__1__)_ = 3.50; SD _(time__1__)_ = 1.18; and Mean _(time__2__)_ = 3.45; SD _(time__2__)_ = 1.08].

Finally, analyses yielded no statistically significant Group by Time interaction with respect to sport sciences students’ views about whether the web [*F*_(__1_,_239__)_ = 0.397; *p* = 0.53; partial eta square = 0.002] and social media [*F*_(__1_,_239__)_ = 0.828; *p* = 0.36; partial eta square = 0.003] represent realistic sources of influence for young people’s attitudes and choices about PAES use.

#### Students’ Self-Efficacy

**FIGURE 2:**
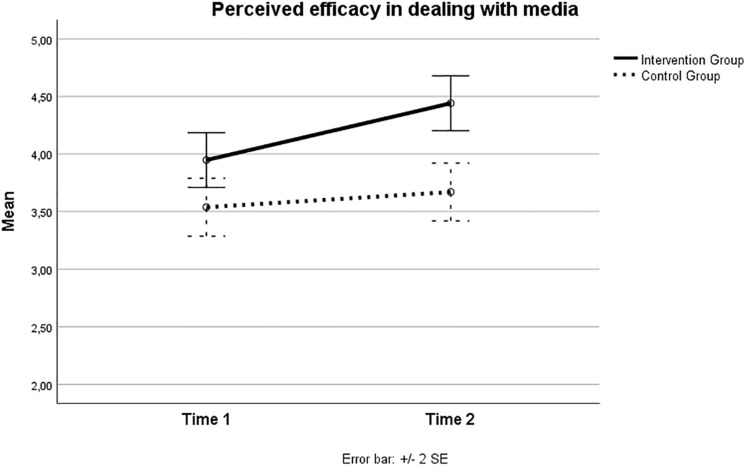
Perceived self-efficacy in dealing with media across time in intervention and control groups.

The results of an analysis of variance (ANOVA) yielded a statistically significant Group by Time effect [*F*_(__1_,_239__)_ = 3.73; *p* = 0.05; partial eta square = 0.015]. Figure 2 sumarizes this interaction effect. As one can see, and as the pairwise comparisons statistically showed, intervention group students had a statistically significant increase over time in their sense of confidence or self-efficacy in dealing with media influence [*F*_(__1_,_239__)_ = 14.66; *p* < 0.001; partial eta square = 0.058]. In contrast, control group students did not, and their self-efficacy remained virtually the same over time [*F*_(__1_,_239__)_ = 0.936; *p* = 0.334; partial eta square = 0.004].

#### Positive Attitudes Toward PAES

Finally, a similar ANOVA conducted on university sport science students’ attitudes toward the use of PAES showed no statistically significant Group by Time interaction effect [*F*_(__1_,_239__)_ = 0.099; *p* = 0.75]. However, a detailed examination of pairwise comparisons yielded a close-to-significance effect among intervention group students [*F*_(__1_,_239__)_ = 3.05; *p* = 0.08; partial eta square = 0.013], whereas this pattern did not emerge for their control group counterpart [*F*_(__1_,_239__)_ = 1.50; *p* = 0.22; partial eta square = 0.006]. Over time, intevention group students showed a reduction in their positive attitudes toward PAES [Mean _(time__1__)_ = 1.81; SD _(time__1__)_ = 0.72; and Mean _(time__2__)_ = 1.69; SD _(time__2__)_ = 1.86], whereas control group students’ attitudes decreased to a lesser extent [Mean _(time__1__)_ = 1.98; SD _(time__1__)_ = 0.86; and Mean _(time__2__)_ = 1.89; SD _(time__2__)_ = 0.84].

## Discussion

This study departed from a broad interest in and scientific attention to the use of controlled and uncontrolled PAES, which tend to characterize both competitive and amateur or fitness sport settings (e.g. Lazuras et al., 2017; Sandvik et al., 2018) and to partly depend on users’ intention to enhance their performance or physical appearance (i.e. PAES – Performance and Appearance Enhancement Substances). PAES use has adverse effects on users’ health, and while this has been clearly established for controlled PAES, such as doping substances (e.g. Bird et al., 2016), adverse effects are emerging for uncontrolled PAES (e.g. Metzl et al., 2001; Biggs et al., 2017). More importantly, PAES use is increasing public policy concerns, as the use of unconrolled PAES (e.g. supplements) may be a “gateway” to more serious use of controlled PAES (i.e. “Gateway Hypothesis,” Lucidi et al., 2008; Zelli et al., 2010; Mallia et al., 2013; Ntoumanis et al., 2014).

Within this broad interest, this investigation mainly focused on the role traditional and current media may have in guiding and shaping young PAES users’ views and choices about these substances. This focus is in line with much scientific evidence pointing to the role of media with respect to several health behaviors, such as smoking, drinking, and dieting (e.g. Austin and Knaus, 2000; Zucker et al., 2008; Scull et al., 2010, 2014). It is also consistent with scholars’ recommendations to implement educational programs of “media literacy”, that is, programs that can promote and foster specific skills whereby young people can correctly interpret and manage media information (Kupersmidt et al., 2010). There currently exists considerable evidence pointing to the beneficial effects media literacy programs have in providing the means and skills to contrast media influence and in reducing risky health behaviors among youth (Se-Hoon et al., 2012; Vahedi et al., 2018).

Lucidi et al. (2017) partly extended this media literacy evidence by showing that high school students’ attitudes toward, and use of, PAES improved as a result of an educational program focusing on the detrimental effects of media. The present investigation moved forward by assessing – among university sport science students – whether a media literacy program broadly focusing on PAES use could be beneficial in fostering specific skills to manage media influence (e.g. awareness of media influence, its perceived realism) and in shaping important individual psychological factors, such as behavioral beliefs, attitudes, self-efficacy, and behaviors. A sample of university sport science students (i.e. intervention sample) actively participated in 18 h of media literacy intervention and provided data prior and after the intervention sessions to assess its efficacy with respect to both media specific outcomes and psychological variables, such as self-efficacy and attitudes. Furthermore, as some literature very recently raised concerns about methodological and measurement issues hindering the possibility of assessing the efficacy of media literacy programs (Potter and Thai, 2019), the present study also relied on a sample of university sport science students who only provided data on the same set of measurements targeted by the intervention.

Data from this latter sample provided a sort of check against the measurements and, as summarized in early sections of the document, the findings indicated non-biased distribution properties of the data and relatively high levels of measurement reliability. All in all, these findings resolved, at least in part, some of the methodological issues and concerns that have been raised (Potter and Thai, 2019).

As to the intervention and its efficacy, the findings of the present investigation offered novel insights on how educational programs focusing on media literacy may provide valid means to counteract the influence media may have on young people’s attitudes and choices toward the use of controlled and uncontrolled PAES. Sport science students’ participation in the intervention sessions had a positive effect, insofar as it contributed to students’ renewed awareness of how media may exert a detrimental effect on PAES use. It also contributed to eliciting among students stronger views about media as “realistic sources” of information and to raise students’ sense of confidence in dealing effectively with the messages and indirect pressure carried by media. These findings are quite in line with other existing research on media literacy training focusing on other substance use behaviors such as, for instance, smoking (Austin et al., 2007).

Sport sciences students obviously are a particularly sensitive at-risk group with respect to PAES use, and these findings are particularly relevant. Sport sciences students are at a higher risk of using uncontrolled, doping, substances than are other university students (e.g. see Thevis et al., 2008), and media literacy needs to be a target of continuing educational efforts. Secondly, sport sciences students are likely to occupy professional positions in sport and educational settings and be coaches, physical trainers, or physical education teachers later in their life. Therefore, it is imperative to ensure they have the necessary skills to correctly interpret, manage, deal with, and warn others about the effects of media messages and influence.

The findings on the efficacy of the media literacy intervention raise additional important considerations. The positive effects on students’ awareness of media influence and on their views about media as “realistic sources” of influence for young people, indirectly suggest that the intervention sessions also fostered students’ ability to analyze media messages, to correctly evaluate their hidden meaning, and to recognize their possible detrimental impact. Findings, however, varied across types of media and, while the above effects were quite clear for traditional forms of media, such as newspapers and TV, they led to weaker conclusions with respect to current forms of media, such as social media and web. In retrospect, it is quite likely that these qualified findings are in part due to the fact that the intervention program heavily relied on media contents and messages drawn primarily from traditional media. Nonetheless, findings solicit the design of media literacy training programs that can more precisely distinguish among these different sources of media influence.

The findings of the investigation also are informative with respect to the value of media literacy training on young people’s social-cognitive experiences. In addition to raising their sense of confidence in dealing effectively with media messages and contents concerning PAES use, the intervention positively affected students by reducing their attitudinal endorsement of PAES use. These findings are in line with, and extend conclusions drawn by, prior research (Lucidi et al., 2017) and by existing meta-analyses conducted with respect to other health behaviors (Vahedi et al., 2018). Furthermore, despite the fact that the intervention was not designed to act directly on sport sciences students’ attitudes, it is fair to speculate that students’ improved skills in dealing with media influence and messages may have positively intervened in shaping students’ beliefs and attitudes toward PAES use.

### Final Considerations

The present investigation has numerous strengths. To date, it represents the first effort addressing directly the efficacy of a PAES media literacy intervention and, in doing so, is the first effort that targeted key media literacy variables both for the intervention and the measurement design. The investigation also focused on a specific population that is quite relevant for the topic of PAES use and the role of media influence (i.e. sport science university students).

These strengths notwithstanding, there exist some limitations that should be noted. First, the investigation was implemented in academic settings and, as a result, the two samples of university sport sciences students were not randomly recruited stratified samples. Therefore, findings should not and can not be generalized to the broader sport science university student population. Secondly, students’ assignment to media intervention or control conditions was not rigorously randomized but, rather, was solely based on students’ willingness to participate in the intervention, thus raising issues of internal validity. Finally, there were methodological concerns that need to be mentioned. Findings might be biased as intervention effects were not evaluated for “contextual effects”, that is, the possibility that students’ individual data would also be a function of the university context in which they were nested (i.e-. students were from four distinct university sites). The small number of universities involved in the investigation could not permit a more rigorous multi-level analysis.

Future studies should extend media literacy interventions to specific high-risk populations (e.g. with a low level of media literacy) and to other settings involving sport sciences professionals and athletes. This broader approach should eventually consolidate and reinforce the value of media literacy programs targeting the use of PAES among youth. Finally, future research also could attempt to impact not only a person’s attitudes, but also his/her emotions, behaviors, and cognitions that result from media exposure (see Cantor and Wilson, 2003; Potter and Byrne, 2007).

## Data Availability Statement

The datasets generated for this study are available on request to the corresponding author.

## Ethics Statement

The studies involving human participants were reviewed and approved by the Ethics Review Board of the Department of Social and Developmental Psychology, University of Rome Sapienza. The participants provided their written informed consent to participate in this study.

## Author Contributions

All the authors have contributed equally and substantially to the development and preparation of the manuscript. Furthermore, all authors have approved the final version of the manuscript. Finally, the authors have agreed to be accountable for all aspects of the manuscript in ensuring that questions related to the accuracy or integrity of any part of it are appropriately investigated and resolved.

## Conflict of Interest

The authors declare that the research was conducted in the absence of any commercial or financial relationships that could be construed as a potential conflict of interest.

## Footnotes

1 Controlled PAES are substances whose use implies legal sanctions (i.e. their use is controlled and regulated by laws) and that are generally indicated as “doping” substances. Thus, these substances are prohibited in competitive sports according to the regulations of the World Anti-Doping Agency (WADA).

2 Uncontrolled PAES are substances that can be freely purchased without any restrictions, and with no legal or social sanctions ensuing from their use (i.e. proteins, creatine, amino-acids, minerals, and vitamins). These substances are generally indicated as “supplements”.
